# Avian toxicoses: a review

**DOI:** 10.3389/fvets.2025.1572736

**Published:** 2025-08-11

**Authors:** Alessandro Vetere, Francesco Di Ianni, Martina Gavezzoli, Ciro Cococcetta

**Affiliations:** ^1^Dipartimento di Scienze Veterinarie e Dell'Allevamento, Università Degli Studi Di Parma, Parma, Italy; ^2^Unité NAC, Centre Hospitalier Vétérinaire Saint Martin, Allonzier-la-Caille, France

**Keywords:** birds, toxicoses, intoxication, avian medicine, exotic pet medicine

## Abstract

Bird species, both domestic and wild, are frequently exposed to a wide range of toxic compounds. Toxicosis in avian patients is often a clinical challenge. Diagnosis often relies on history and clinical signs because specific tests for most toxicants are not available in clinical practice, or they require unsustainable blood volume for small avian species. The management of toxicoses consists of patient stabilization, decreasing toxin absorption, improving its elimination, and supporting therapy. This literature review provides an outline of the most common toxicoses in birds, as well as their diagnosis and possible treatments.

## 1 Introduction

Toxicoses are relatively common in pet birds (Table 1), and the route of intoxication can vary, including ingestion, inhalation, or transcutaneous absorption. Indeed, several cases have been reported in the literature concerning both heavy metal intoxication and toxic plant ingestion (1–3). Birds are smart and curious animals, and the accidental risk of toxic substance ingestion is relatively high. The physiological response to toxic agents is highly variable and influenced by numerous factors. These include interspecies and individual differences, body weight and size, nutritional status, age, and sex. Additionally, the nature of the specific toxicant, the route of exposure, and the quantity of the toxin inhaled or systemically absorbed play critical roles in determining the overall toxicological impact (3). In the case of acute poisoning, appropriate therapy depends on the type of poison and the route of toxin absorption and distribution. Birds that have only very recently been in contact with toxic agents may be helped by topical flushing with warm water or sterile saline for at least 20–30 min (4). Additionally, the induction of vomiting is not recommended in cases of poison ingestion because of the likely chance of aspiration pneumonia (*ab-ingestis*) (5, 6). In cases of ingestion occurring shortly before symptom presentation (within a 1- to 3-h window), delicate flushing of the crop with warm water or saline while keeping the patient under general anesthesia and artificially ventilated via tracheal tube is suggested (5, 6). The procedure is contraindicated if the ingestion was of oily or caustic material (5). Moreover, the oral administration of activated charcoal at a dosage of 1–3 g/kg of body weight has been reported to be useful as a toxin adsorber (7). Additionally, osmotically active substances such as psyllium and sorbitol have been used (8).

**Table 1:** A list of some toxic substances described as toxic for birds, as well as their effects, diagnosis, and possible therapy.

**Toxic compound**	**Common clinical signs**	**Diagnosis**	**Therapy**
Alcohol (ethanol)	Incoordination, neurological symptoms	Toxicologic examination on crop's content, blood, and liver	Not reported. Supportive care should be considered.
Anticoagulant rodenticides	Weakness, lethargy, pale mucous membranes, bleeding from orifices, and fatal hemorrhage	Clinical history	Decontamination, supportive care, Vit K1 IM until stabilization, and then PO every 24 h for 3 weeks
Avocado (*Persea americana*)	Gastrointestinal signs (vomiting, diarrhea, and abdominal pain).	Clinical history	Supportive care
Barbiturates	CNS depression, coma, death (10, 62), vomiting, regurgitation, and diarrhea	Clinical history, barbituraemia	Supportive care
Chocolate	Vomiting, diarrhea, and death	Clinical history	Supportive care, such as fluidotherapy, oxygen, and antiarrhythmic drugs
Diclofenac	Lethargy, weakness, anorexia, and neurological symptoms	Clinical history	Supportive care
Heavy metals [lead (Pb), mercury (Hg), copper (Cu), cadmium (Cd), zinc (Zn), arsenic (A*s)]*	Depression, CNS signs, regurgitation, diarrhea, and anorexia. Neurological signs include weakness, tremors, seizures, disorientation, or altered behavior. Heavy metal toxicity can affect the kidneys, thus leading to polyuria, polydipsia, dehydration, or renal failure	Physical examination, history of metal exposure, blood tests, and radiology may be used to detect the presence of metal in the gastrointestinal tract	If present in the gastrointestinal tract, physical removal of a metallic foreign body. Injectable chelating agents such as calcium disodium ethylenediamine tetraacetate (Ca EDTA) 35 to 40 mg/kg IM every 12 h for 5 days and repeated if needed Dimercaptosuccinic acid given at 25 to 35 mg/kg PO q12h for 7 to 10 days or at 25 to 35 mg/kg PO q12h for 5 days a week for 3 to 5 weeks. Doses of 80 mg/kg or higher are lethal. D-penicillamine is another oral chelator that is given at 30 to 55 mg/kg PO q12h for 1 week, stopped for 1 week, and repeated if needed.
Hydrocarbons	Respiratory signs (coughing, wheezing, and labored breathing) or gastrointestinal symptoms (vomiting and diarrhea)	Clinical history	Supportive care
Melaleuca oil (Tea tree oil)	Vomiting and coma	Clinical history	Supportive care
Mycotoxins	Lethargy, weight loss, anorexia, regurgitation, and polydipsia	Clinical history	Supportive care, glucomannans, and organic selenium as nutritional supplementation
Smoke	Dermatitis, sinusitis, and aerosacculitis	Clinical history	Supportive care
Sodium chloride (NaCl)	Apathy, incoordination, opistotonus, and dyspnoea	Clinical history	Supportive care
Polytetrafluoroethylene (Teflon)	Dyspnoea seizures, weakness, coma, and death	Clinical history	Oxygen therapy, bronchodilators, NSAIDs, diuretics, and antimicrobials agents
Urea	Anorexia, ataxia, vomiting, diarrhea, convulsions, and death. Neurological symptoms can include depression, weakness, disorientation, tremors, seizures, or ataxia. Urea toxicity can affect the digestive system, thus leading to symptoms such as vomiting, diarrhea, loss of appetite, and weight loss	Hematology and clinical history	Fluid therapy and supportive care
Vitamin A	Swollen eyes, conjunctivitis, and excessive tearing and clouding of the cornea. Skin can be prone to dryness and cracking	Clinical history, vitamin A concentration in plasma, and liver samples	Supportive care, vitamin A discontinuation in case of over supplementation
Vitamin D	Depression, anorexia, vomiting, polyuria and polydipsia, joint pain, muscle weakness, and soft tissue mineralization	Clinical history, vitamin D, calcium, and phosphorus levels	Supportive care and hydration (10). Furosemide in cases of persistent hyper-calcaemia

## 2 Inclusion criteria

A literature search was conducted in the databases Google Scholar, PubMed, and Scopus using the following keywords: “bird intoxication,” “toxicosis in birds,” and “nocive compounds for birds.” Only peer-reviewed articles were included. Publications that did not meet this criterion or were not relevant to the aims of the review were excluded.

## 3 Polytetrafluoroethylene (PTFE)

In pet birds, deadly polytetrafluoroethylene (PTFE, Teflon^TM^) fume intoxications are not uncommon. PTFE is an inert, synthetic fluoropolymer of tetrafluoroethylene that is commonly used on cookware and heat bulbs, among other materials, as a scratch-resistant and waterproof agent (1, 9, 10). When PTFE is heated above 280°C, it decomposes into particulates and fluorinated acid gases, such as carbonyl fluoride, hydrogen fluoride, and perfluoroisobutylene, which are extremely toxic to birds when inhaled (1, 2). Smaller birds, such as cockatiels and budgerigars, seem to be more sensitive than larger birds (11). Clinical signs include dyspnoea, incoordination, weakness, coma, and death (1, 2). Moreover, the toxic effects of these gases on the myocardium lead to arrhythmias and cardiac failure. On necropsy, pulmonary oedema and hemorrhages are common findings (2, 12). Furthermore, treatment consists of oxygen therapy, bronchodilators, non-steroidal anti-inflammatory drugs (NSAIDs), diuretics, and antimicrobial agents; however, the prognosis is often poor (1, 10).

## 4 Smoke

Smoke toxicosis in birds occurs when they are exposed to harmful gases, particulate matter, and toxins produced during fires or exposure to smoke. Such incidents can have severe consequences on avian health, thus leading to respiratory distress, systemic toxicity, and even mortality. Smoke toxicosis in birds primarily arises from exposure to smoke generated by wildfires, structural fires, or even indoor cooking accidents (13). Gasleaks from the heating system in birds kept indoors in temperate climates can be highly dangerous, as these animals are far more sensitive to such toxins than humans. The composition of smoke varies depending on the materials that are burned, such as wood, plastics, or chemicals, and can include carbon monoxide (CO), nitrogen dioxide (NO2), Hydrogen cyanide (HCN), volatile organic compounds (VOCs), and respirable particulate matter. Additionally, exposure to smoke can have a range of adverse effects on birds' respiratory, cardiovascular, and neurological systems. The inhalation of toxic gases and particulate matter can cause thermal damage, irritation, and inflammation of the respiratory tract, thus leading to difficulty breathing, coughing, wheezing, and nasal discharge, progressing to sloughing, edema, and airway obstructions.

Systemic effects vary depending on the inhaled toxin and may include hypoxia due to carbon monoxide binding to hemoglobin or disruption of cellular respiration caused by cyanide ions. These mechanisms result in impaired oxygen delivery to tissues or the inability of tissues to utilize oxygen, leading to subsequent tissue damage (5, 14). Birds may also exhibit lethargy, weakness, reduced appetite, and neurological symptoms, such as disorientation or seizures (13). The severity of the symptoms depends on the duration and intensity of exposure, as well as the species, age, and overall health of the bird (13). The immediate management of smoke toxicosis in birds involves removing them from the smoke source and providing a clean and well-ventilated environment (1, 10). Supportive care may include oxygen supplementation, fluid therapy, nebulization, and bronchodilators to alleviate respiratory distress and promote healing of the respiratory tract (1). In addition, anti-inflammatory medications can be administered to reduce airway inflammation, and antioxidants may help to counteract the oxidative stress induced by smoke exposure. Hyperbaric oxygen therapy should always be considered if available. Long-term follow-up and monitoring are essential to assess the recovery of affected birds and to identify potential complications (1, 10).

## 5 Hydrocarbons

Hydrocarbon toxicosis in birds occurs when they are exposed to petroleum-based products, such as oil spills, industrial waste, or accidental ingestion of household substances (15). Hydrocarbon toxicosis can have deleterious effects on the avian respiratory, gastrointestinal, and nervous systems, as well as cause feather damage. The clinical effects of hydrocarbon exposure depend on the volatility, chain length, and presence of potential contaminants in the specific hydrocarbon. Inhalation or ingestion of hydrocarbons can lead to respiratory distress, such as coughing, wheezing, and labored breathing (10, 16). Moreover, gastrointestinal symptoms may include vomiting, diarrhea, and loss of appetite. Hydrocarbons can also have neurotoxic effects, thus causing disorientation, seizures, or impaired coordination (10, 15, 16). The severity of symptoms depends on the species, age, and health status of the birds, as well as the concentration of hydrocarbons (10). Immediate measures include removing the bird from the source of exposure, preventing further ingestion or inhalation of hydrocarbons, and providing a clean and quiet environment (10). Furthermore, decontamination involves careful washing of the bird's feathers with mild detergents specifically designed for wildlife. In cases of heavy hydrocarbons, an initial treatment with an oil-based solvent is often necessary to effectively remove the material. It has also been reported that the use of margarine is to remove polyisobutylene in wild Anhinga (*Anhinga anhinga*) and Great Blue Heron (*Ardea herodias*) (17).

Crop lavage is highly discouraged in cases of ingestion, as the risk of aspiration during this procedure is too high. Veterinarians may administer supportive care, such as fluids, nutrition, and anti-inflammatory drugs, to address respiratory distress or other symptoms. Long-term monitoring is necessary to assess the recovery of affected birds and potential complications (10).

In general, clinical signs depend on various factors, such as the nature of the toxin, route of exposure, amount of toxin absorbed, and whether the poisoning is acute or chronic (18).

## 6 Heavy metals [lead (Pb), mercury (Hg), copper (Cu), cadmium (Cd), zinc (Zn), and arsenic (As)]

Heavy metal poisoning is a significant environmental concern affecting avian species worldwide. Several heavy metals can pose a threat to avian health. The most encountered heavy metals in bird poisoning cases include lead (Pb) and zinc (Zn), while other metals such as mercury (Hg), cadmium (Cd), copper (Cu), and arsenic (As) are less frequently observed (5, 19–21). Birds can be exposed to heavy metals through various routes: in domestic animals, intoxication often occurs through the ingestion of metal objects or continuous consumption of small parts of cage bars with metal-based paints. In the wild, birds may be exposed to heavy metals through the consumption of contaminated food or water, as well as the ingestion of lead-based ammunition, fishing tackle, or discarded materials containing heavy metals, all of which can lead to poisoning (5). Clinical signs of heavy metal poisoning in birds can vary depending on the type and level of metal exposure. Common symptoms involving the gastrointestinal system include regurgitation, diarrhea, and anorexia. Additionally, neurological signs can include weakness, tremors, seizures, disorientation, or altered behavior. Heavy metal toxicity can affect the kidneys, thus leading to polyuria, polydipsia, dehydration, or renal failure (1, 10).

In all birds, zinc toxicosis and lead toxicosis share many clinical signs (anorexia, gastrointestinal stasis, weakness, neurologic deficits, dehydration, and anemia). Distinguishing features can be subtle: lead poisoning more often causes dark-green droppings (from bile pigments), hemoglobinuria, severe neurological deficits (e.g., wing drop in raptors), and anemia; while zinc poisoning tends to produce extreme polyuria/polydipsia, pancreatic-kidney damage, and occasionally unusual signs such as feather loss or cyanosis (22).

Based on the findings of Martel et al., lead intoxication should be considered a differential for acute-onset paralysis of the distal pelvic limbs in psittacine birds (23). In those three cases, blood lead levels were higher than the normal limit. However, despite achieving persistent reduction of blood lead levels following parenteral chelation therapy using calcium disodium ethylenediamine tetraacetate (EDTA), all three birds had continued neurological deficits in both pelvic limbs. Birds were considered to maintain a good quality of life after husbandry changes were implemented to assist in ambulation.

The diagnosis of heavy metal poisoning in birds involves a comprehensive approach that includes a physical examination, assessment of weight, history of metal exposure, observation of symptoms, and various diagnostic tests (10, 11). Blood tests can be conducted to assess liver and kidney function through biochemical parameters and the presence of anemia by evaluating hematocrit levels. It is also possible to measure heavy metal levels in the blood, serum, or plasma (depending on the metal being tested), which can indicate the presence of lead or zinc toxicity, thus aiding in the definitive diagnosis. Radiology may be used to detect the presence of metal in the gastrointestinal tract. Additionally, your veterinarian will evaluate your bird for signs of crop stasis, which can be an indication of lead poisoning (1, 10). Initial treatment includes supportive therapy and, if applicable, surgical or endoscopic removal of the metallic foreign body. Chelation therapy is provided through parenteral administration (intramuscular [IM] or subcutaneous [SC] injection) of chelating agents, such as calcium disodium ethylenediamine tetraacetate (Ca EDTA) at a dosage of 35–40 mg/kg every 12 h for 5 days, with repetition if needed. Oral administration of chelating agents is possible using dimercaptosuccinic acid (DMSA/succimer) and D-penicillamine.

Dimercaptosuccinic acid is a chelator that can decrease lead plasma levels and is given at 25 to 35 mg/kg PO q12h for 7 to 10 days or at 25 to 35 mg/kg PO q12h for 5 days a week for 3 to 5 weeks. Doses of 80 mg/kg or higher are lethal (13, 24–29).

CaNa2EDTA (the preferred initial chelator) and DMSA are both effective, but the DMSA range of safety is smaller (13, 25).

D-penicillamine is another oral chelator that is given at 30 to 55 mg/kg PO q12h for 1 week, stopped for 1 week, and repeated if needed with or without CaNa2EDTA (25, 28). The main adverse effect is gastrointestinal upset (13, 24, 28).

Long-term chelation can deplete zinc, iron, and/or manganese, so these values should be monitored during and after chelation. Chelation therapy has several other side effects and should be administered with monitoring of the side effects, best keeping the birds hospitalized (5, 19, 20, 22).

Cadmium and mercury toxicosis can be treated with CaNa2EDTA and with DMSA, vitamin E, and/or selenium (13, 25).

## 7 Tea tree oil

Tea tree oil (*Melaleuca alterniflia*) intoxication in a pet cockatiel (*Nymphicus hollandicus*) has also been reported in a case report (30). In this report, the cockatiel was presented for clinical examination due to a serious despondency that occurred after 3 drops (0.15 ml) of tea tree oil (*Melaleuca alterniflia*) were directly applied to the cutis; before entering into a comatose state, the parrot suffered convulsions and vomiting that lasted approximately 15 min. The symptoms appeared 30 min after the essential oil was applied. The animal was discharged after 48 h of hospitalization in stable condition and after supportive therapy. *Melaleuca alternifolia*, which is otherwise known as the “Tea tree,” is an Australian species from the northern coast with a high content of terpinen-4-ol (more than 30% of gross weight) and a low content of cinenol (lower than 15%) (31). Terpenes are a class of organic compounds produced by a variety of plants (32) and animals, such as some insects (33). There have been some studies about the toxicity of *Melaleuca alternifolia* and its extract (30, 34–36); however, only a few reports about the toxicity of other terpenes and their metabolites have been published (36, 37). Regarding the toxicity of terpenes in pet birds, scientific literature is lacking.

## 8 Sodium chloride (NaCl)

Sodium chloride (NaCl) is frequently applied to roads, especially in winter, to melt ice and snow and prevent slippery conditions. Due to the difficulty in recovering bird carcasses and frequent underreporting, the true extent of mortality caused by road salt is likely underestimated. Most documented cases involve winter finches (subfamily *Carduelinae*), likely due to their diet, their presence in snow-covered regions, and their tendency to form large, more detectable flocks. Their strong attraction to salted roads suggests that the ecological impact of road deicing practices is greater than previously recognized, and the role of salt as a direct mortality factor has likely been underestimated by wildlife and transportation authorities (38). A suspicion of acute sodium chloride intoxication appeared in three different broiler farms involving chicks from 10 days to 30 days of age (39). The chicks presented with severe depression, apathy, and CNS symptoms, such as incoordination, opisthotonos, and dyspnoea after the ingestion of feed with a sodium chloride content between 0.4% and 0.9%. Pathological findings included swollen oedematous shanks, extensive subcutaneous oedema, hydropericardium, pale muscles, pale kidneys, and ascites (39). The clinical signs and mortality observed in broiler farms were strongly associated with the levels of salt or sodium present in the feed, as well as the age of the flocks. Younger flocks exhibited more severe and acute symptoms, thus leading to higher mortality rates (up to 20%). Another study described the toxic effects of NaCl on house sparrows (*Passer domesticus*). Lethargy, poor coordination, inability to fly, and death appeared as early as 30 min after exposure at doses of 1,500 mg/kg and above (40). High plasma sodium levels (≥200 mmol/l) were consistently associated with these clinical signs. Pathological examination revealed fluid accumulation and swelling in the gizzard at higher doses, while no structural brain damage was observed despite significantly elevated brain sodium concentrations. As there is no specific antidote for sodium chloride toxicity in birds, supportive care is the main approach (41). Intravenous or subcutaneous fluids may be necessary to dilute elevated plasma sodium levels. If neurological symptoms such as ataxia or seizures are present, hospitalization and careful monitoring of electrolytes are required (41).

## 9 Alcohol (ethanol)

Alcohol (ethanol) exerts its toxic effects primarily through central nervous system depression, metabolic disruption, and cellular damage. It enhances GABA activity and inhibits glutamate in the brain, leading to sedation, impaired coordination, and, at high doses, respiratory depression or coma (42). In the liver, ethanol is metabolized to acetaldehyde, then to acetic acid. This process increases the NADH/NAD^+^ ratio, disrupting normal metabolism and causing lactic acidosis, hypoglycemia, ketoacidosis, and fatty liver (42). Ethanol is a natural byproduct of fruit sugar fermentation and has likely co-evolved with frugivorous animals since the emergence of fleshy fruits. It has been proposed that frugivores might use the scent of ethanol to locate fruiting plants and therefore prefer fruits with higher ethanol content. One study tested this hypothesis in three frugivorous bird species, the Cape white-eye (*Zosterops virens*), speckled mousebird (*Colius striatus*), and red-winged starling (*Onychognathus morio*), and found no significant preference for artificial fruit containing 1% ethanol compared to ethanol-free fruit. These findings suggest that, contrary to earlier assumptions, birds do not show a behavioral preference for high-ethanol fruits, even at concentrations typically found in overripe fruit (43).

Stephen LJ and colleagues reported on an acute case of intoxication in a group of 16 wild Bohemian waxwings (*Bombycilla garrulus)* and one pine grosbeak (*Pinicola enucleator*) that ate fermented crab apples. The birds exhibited neurological signs, such as incoordination, that led them to crash into the nearby hospital's windows (44). Necropsy was performed, and death was attributed to internal hemorrhages secondary to blunt trauma against the windows. However, gizzards from the 17 birds containing crab apple mash and pooled blood were analyzed. A total of 3.1 g alcohol/100 ml mash and 73 mg alcohol/100 ml blood were detected. At these dosages, a presumptive diagnosis of acute alcohol intoxication was made. Another report by Fitzgerald SD and colleagues (40) describes a suspected ethanol intoxication in two cedar waxwings (*Bombycilla cedrorum*) that died acutely after feeding on fermented hawthorn pommes. Crop contents and liver were collected for toxicological evaluation. The crop content and liver ethanol levels supported the diagnosis of ethanol toxicosis (45).

In pet birds, such as parrots, cases of accidental alcohol ingestion have been reported, sometimes even following deliberate offering by the owner, as documented in videos shared on social media. Depending on the amount ingested, affected birds may show impaired flight and coordination. Since these animals are not prey species and are kept in protected environments, it is likely that in cases of small-dose exposure, clinical signs resolve spontaneously without causing permanent harm (46).

Treatment of choice involves supportive fluid therapy and keeping the animal in a dark, quiet environment, ensuring it cannot harm itself.

## 10 Urea

Urea is a nitrogen-containing compound produced in the liver as the main end-product of protein metabolism in mammals. It is excreted in urine and widely used in agriculture as a non-protein nitrogen (NPN) source in ruminant feed to promote microbial protein synthesis in the rumen (47). Urea-based toxicosis is common in broiler farms, but it can rarely occur in cases of feeding errors in backyard poultry or significant hygiene issues in indoor bird husbandry. In birds, urea intoxication can result from various factors, including dietary imbalances or exposure to urea-based fertilizers or other environmental sources (18, 47). In Galliformes, urea intoxication due to fertilizer ingestion (18) was described. Excessive levels of urea can have toxic effects on bird physiology and health. Urea intoxication disrupts normal metabolic processes and can lead to various symptoms, including neurological symptoms, such as depression, weakness, disorientation, tremors, seizures, or ataxia. Urea toxicity can affect the digestive system, thus leading to symptoms, such as vomiting, diarrhea, loss of appetite, and weight loss. High levels of urea in the bloodstream can impact lung function, thus leading to difficulty breathing or respiratory distress. Diagnosis relies on hematology and clinical history. In a recent study (47), broilers exposed to intoxication showed a significant reduction in various hematological parameters, including erythrocytic and total leucocytic counts, hemoglobin (Hb), and packed cell volume (PCV). These effects were observed in a dose- and time-dependent manner. Additionally, the biochemical profile of the intoxicated birds demonstrated a significant decrease in blood glucose levels and a significant increase in serum uric acid, urea, alkaline phosphatase (ALP), and lactate dehydrogenase (LDH) levels compared to the control levels. In ruminants, the oral administration of acetic acid or vinegar helps lower the ruminal pH, converting toxic ammonia (NH3) into the less absorbable ammonium ion (${\text{NH}}_{4}^{+}$). Cold water can also be administered to slow microbial urease activity and reduce ammonia production (48). In birds, as with other toxicoses, supportive and nutritional care are recommended (5, 10).

## 11 Methylxanthines

Methylxanthines are a group of compounds that include caffeine, theobromine, and theophylline. Birds that are free to wander in domestic environments can deliberately feed on toxic plants or toxic foods (such as chocolate and coffee) containing metilxantines (49–52). Methylxanthines, such as caffeine, theobromine, and theophylline, exert their toxic effects in birds primarily by acting as adenosine receptor antagonists, which leads to central nervous system stimulation, tachycardia, arrhythmias, increased blood pressure, diuresis, and relaxation of smooth muscles, including bronchial muscles. Additionally, they inhibit phosphodiesterase, resulting in increased intracellular cyclic AMP (cAMP), which further amplifies their stimulatory effects (53). In birds, these substances are rapidly absorbed, and due to their small body size and high metabolic rate, even small amounts can cause severe clinical signs, including hyperactivity, tremors, seizures, and potentially sudden death (50, 51). Common symptoms of methylxanthine toxicity in birds may include restlessness, increased activity levels, increased heart rate, muscle tremors or seizures, and gastrointestinal symptoms, such as vomiting and diarrhea (50, 51, 53). Diagnosis of methylxanthine intoxication in birds is primarily based on a sudden onset of neurological and cardiac signs combined with a history of exposure to substances such as chocolate, coffee, or caffeine-containing products. Laboratory confirmation is rarely performed but may involve detection of methylxanthines in blood or tissues. In most cases, diagnosis is clinical and presumptive, supported by observed symptoms and known or suspected ingestion (50–52). The few reported cases of chocolate toxicosis in birds resulted in death, except for a case of a common mynah (*Acridotheres tristis*) (50–52). In this case, diphenhydramine HCl elixir was prescribed, and the mynah bird completely recovered in 4 days. A deadly chocolate intoxication was also documented in a kea (*Nestor notabilis*) specimen (49). If the onset is recent, decontamination should be performed. Crop lavage is indicated if the toxin has been recently ingested. Supportive care, such as fluid therapy, oxygen, antiarrhythmic drug, and sedative drugs (benzodiazepines) administration, should be performed as needed (10).

## 12 Mycotoxins

Mycotoxins are toxic secondary metabolites produced by fungi such as *Aspergillus, Fusarium*, and *Penicillium*, and they commonly contaminate feed and food products (54–59). Their toxic effects vary depending on the type of mycotoxin, dose, exposure time, and the species affected (59). One of the main toxic mechanisms is the inhibition of protein synthesis, particularly by trichothecenes such as T-2 toxin and deoxynivalenol, which disrupt ribosomal function and lead to cell death, especially in rapidly dividing tissues, such as the intestinal lining and bone marrow. Many mycotoxins, including aflatoxins and ochratoxin A, also induce oxidative stress by generating reactive oxygen species, causing lipid peroxidation, mitochondrial dysfunction, and damage to DNA and proteins. Immunosuppression is another major consequence, as toxins, such as aflatoxins, fumonisins, and ochratoxin A, impair the function of lymphocytes and macrophages, reducing resistance to infections and vaccine efficacy. Aflatoxins are known for their hepatotoxic effects, leading to liver damage and increased risk of hepatocellular carcinoma, while ochratoxin A primarily causes kidney damage. Some mycotoxins, such as fumonisins, have neurotoxic properties due to their disruption of sphingolipid metabolism, leading to neurodegenerative diseases, such as equine leukoencephalomalacia. Others, such as zearalenone, act as endocrine disruptors by mimicking estrogen, resulting in reproductive disorders in animals. Additionally, aflatoxins are genotoxic and carcinogenic, forming DNA adducts and causing mutations, especially in the TP53 tumor suppressor gene (58, 59, 59). Avian species are more susceptible than other affected species, such as dogs, cattle, swine, and humans, to aflatoxicosis (54). Clinical signs vary depending on the type and dose of the mycotoxin. However, they may include reduced feed intake, poor growth, diarrhea, immunosuppression, neurological symptoms (in the case of tremorgenic toxins), reproductive issues, such as decreased egg production or fertility, and sudden death in severe cases (54, 55). _Aflatoxin and fusariotoxin are often responsible for avian mycotoxicosis and are usually associated with cereal grains, corn, and peanuts that have been exposed to or maintained in humid, moist conditions (54). In aflatoxicosis, the primary target organ of damage is the liver; clinical signs of chronic aflatoxicosis often include lethargy, weight loss, anorexia, regurgitation, and polydipsia (54, 56). Fusariotoxicosis is associated with mucosal necrosis. Although there is no specific treatment for mycotoxicosis, birds that are at high risk of exposure may benefit from supplementation with glucomannans and organic selenium, which appear to decrease the hepatotoxic and CNS changes associated with exposure (57, 58). Diagnosing mycotoxin intoxication in birds is often difficult due to the non-specific nature of clinical signs, which can mimic infectious diseases, nutritional deficiencies, or other toxicities. Diagnosis typically relies on a combination of factors, including clinical observation, flock history, necropsy findings, and feed analysis (1, 5, 52, 60–64). Suspicion usually arises when multiple birds in a group show signs after consuming moldy or poorly stored feed, particularly under humid conditions. Necropsy may reveal enlarged, pale, or hemorrhagic livers (commonly seen with aflatoxicosis), kidney damage (associated with ochratoxin A), or gastrointestinal lesions (65). Histopathology can show hepatocellular necrosis, bile duct proliferation, or renal tubular degeneration (65). Definitive diagnosis requires laboratory testing of feed, most commonly using ELISA or chromatographic methods (HPLC, LC-MS/MS) to detect specific mycotoxins (66). Differential diagnoses must rule out common avian infections, heavy metal poisoning, and nutritional disorders. Ultimately, an accurate diagnosis of mycotoxicosis in birds depends on the integration of clinical signs, pathological findings, and confirmation of toxin presence in feed.

## 13 Cyanobacterial and algal

Harmful algal blooms (HABs), increasingly frequent due to climate change and eutrophication, are recognized as a significant source of morbidity and mortality in wild and captive avian species. These blooms can produce potent toxins, such as microcystins, saxitoxin (STX), and domoic acid (DA), which may bioaccumulate in aquatic ecosystems and pose serious health threats to birds (67).

Microcystins, hepatotoxins produced by freshwater cyanobacteria, have been implicated in fatal outbreaks among captive American white pelicans (*Pelecanus erythrorhynchos*) housed in a spring-fed pond. Over a 5-month period, seven out of eight pelicans died or were euthanized after presenting with clinical signs, including anorexia, weight loss, and inability to stand. Laboratory findings revealed heterophilic leukocytosis and elevated muscle enzymes, while histopathology showed severe, chronic rhabdomyofiber degeneration and necrosis. Despite routine vitamin E supplementation, the findings were consistent with microcystin toxicosis based on toxin detection in tissues and pond water (68). The authors hypothesized that oxidative stress and vitamin E depletion may have exacerbated muscle degeneration and cardiomyopathy in affected birds.

In marine environments, neurotoxic HABs producing STX and DA have also been associated with seabird die-offs. Between 2014 and 2017, widespread mortality events were reported in the Bering and Chukchi seas, affecting mainly northern fulmars (*Fulmarus glacialis*) and short-tailed shearwaters (*Ardenna tenuirostris*). STX was detected in 60% of tested individuals and in 88% of northern fulmars, with toxin concentrations within the range of those found in previous STX-related avian mortality events. Although starvation appeared to be the proximate cause of death, exposure to STX likely contributed to the observed clinical deterioration (69).

A broader retrospective study examining seabird carcasses collected between 2007 and 2018 across the U.S. coastal regions found domoic acid in 70% and saxitoxin in 23% of cases. DA was frequently detected in gastrointestinal contents, liver, bile, and kidney, while STX was most commonly found in liver and bile. In some cases, both toxins were present simultaneously, suggesting co-exposure and complex interactions in toxicosis pathways (69).

The growing body of evidence linking HAB-related toxins to avian mortality highlights an urgent need for improved surveillance, diagnostic capabilities, and ecological research. Understanding sublethal effects, chronic exposure risks, and species-specific sensitivity is essential for anticipating the impact of algal toxin events on avian populations and implementing preventive strategies.

## 14 Rodenticides

Rodenticides are chemical compounds that are used to control rodent populations. Unfortunately, these substances can have unintended consequences for non-target wildlife, including birds. Rodenticide toxicosis in birds occurs when they are exposed to or consume rodenticides either directly or through the ingestion of contaminated prey. Rodenticide intoxication is not uncommon in birds of prey (29, 70) after the ingestion of rats or mice poisoned by warfarin or brodifacoum (24, 70). Most rodenticides exert an anticoagulant effect by interfering with blood clotting mechanisms, especially by inhibiting vitamin K epoxide reductase, thus causing a depletion of active vitamin K and leading to uncontrolled bleeding (71). The severity of symptoms depends on the species, age, health status, and quantity and duration of rodenticide exposure. Second-Generation Anticoagulant Rodenticides are slowly metabolized by the liver and can persist for at least 6 months in liver and tissues (72). Birds may exhibit symptoms, such as weakness, lethargy, pale mucous membranes, bleeding from orifices, and bruising (10). As there is little information about clotting profiles in avian medicine, diagnosis is often made postmortem (10). In cases of recent exposure, decontamination and supportive care are crucial to prevent further absorption of the toxic substance. If ingestion occurred recently, crop lavage is indicated. Vitamin K1 is commonly used as an antidote for anticoagulant rodenticide toxicosis in birds (2.2 mg/kg q for 4 h to 8 h), administered IM until stabilization and then PO every 24 h for 3 weeks (73). This treatment helps to restore normal blood clotting mechanisms by replenishing the depleted vitamin K-dependent clotting factors. In severe cases of rodenticide intoxication resulting in significant blood loss, blood transfusions may be necessary to replace lost blood components and to support bird recovery (10, 74). The severity of symptoms depends on the species, age, health status, and quantity and duration of rodenticide exposure (75). Non-anticoagulant rodenticides, such as zinc phosphide or bromethalin, affect the central nervous system and can cause neurologic symptoms, including tremors, seizures, and ataxia. Bromethalin toxicosis in 14 feral conures (Psittacara species) has been reported. The parrots showed different degrees of paraparesis, with some cases progressing to dysphagia, ataxia, and tetraparesis. Histopathologic lesions included astrogliosis, pallor, and vacuolation of white matter in the brain, mostly in the medial longitudinal fasciculus, pons, optic tectum, cerebellar peduncle, and ventral funiculus. The neuropathological changes noted in these conures were similar to those of mammals exposed to bromethalin, but with a characteristic distribution that was speculated to be associated with a higher susceptibility to cytotoxic edema in certain regions of the avian brain. Bromethalin toxicosis should be considered in the differential list for any avian patient presenting with neurologic disease and potential exposure to a rodenticide (76).

A recent and innovative application of advanced chromatographic techniques coupled with tandem mass spectrometry has demonstrated high diagnostic potential for environmental monitoring of pesticide contamination. In this study, 108 pesticides and their metabolites—including several persistent organic pollutants (POPs)—were detected and quantified in muscle samples collected from 25 deceased birds of prey (families Accipitridae, Falconidae, and Strigidae) admitted to a rehabilitation center in Madrid, Spain, in 2013. Notably, several banned pesticides were identified at high concentrations, highlighting ongoing environmental contamination. Given the exceptional sensitivity and analytical performance of these chromatographic methods, they represent a valuable alternative to traditional approaches for ecotoxicological and forensic investigations. Persistent organic pollutants, such as chlorinated pesticides, polychlorinated biphenyls (PCBs), and polycyclic aromatic hydrocarbons (PAHs), are of particular concern due to their environmental persistence, long-range transport, bioaccumulation, and known adverse effects on both wildlife and human health (77).

## 15 Avocado (*Persea americana*)

In psittacine, avocado (*Persea americana*) intoxication has also been reported (8). The exact toxic component of avocado that is responsible for its effects on birds is not well understood. However, it is believed that the compound persin, which is found in various parts of the avocado (including the leaves, bark, skin, and pit), is the primary toxic agent (54, 78). Persin is a fatty acid derivative and is known to be toxic to some animals, although the sensitivity to persin can vary among species (78, 79). Necropsy findings may include lung and liver congestion, pulmonary and subcutaneous oedema, and pericardial and/or coelomic effusion. Myocardial degeneration with heterophilic infiltration is a common histopathological finding in ostriches (10, 80). Moreover, symptoms may include difficulty breathing, increased respiratory rate, weakness, depression, anorexia, and sudden death (10, 54, 80). Gastrointestinal signs, such as vomiting, diarrhea, and abdominal pain, may also occur. The severity of symptoms can vary depending on the amount of avocado ingested and the individual bird's susceptibility (54). As there are no specific tests available to confirm the diagnosis of avocado intoxication, it relies on a history of exposure and clinical signs. If recently ingested, treatment may include administering activated charcoal to minimize further absorption of the toxic components. Furthermore, supportive care aims to alleviate symptoms and maintain the bird's overall health. This may involve providing supplemental oxygen, fluid therapy to maintain hydration, and nutritional support (10, 80).

## 16 Toxic plants (miscellaneous)

There have been a few reports of pet bird plant toxicosis; however, these individuals are likely to be exposed to plants in their environment. Many ornamental plants are toxic, such as *Coronilla varia, Ericaceae* family (for example *Rhododendron spp*.), *Kalanchoe spp*., *Nerium oleander, Taxus media, Digitalis purpurea, and Euphorbia pulcherima* (81). Ericaceae contains greyanotoxin, which causes seizures, ataxia, paralysis, coma, and even cardiac effects. Moreover, taxus, oleander, and digitalis cause cardiac toxicities. *Kalanochoe* spp. are kept as houseplants for their colorful flowers and ease of care, and they can cause ataxia, depression, muscle tremors, seizures, paralysis, and death. Plants that contain tannic acid have been reported to be hepatotoxic (82). Additionally, *Euphorbia pulcherrima* can induce gastrointestinal signs (Table 2).

**Table 2:** Some plants are toxic to pet birds.

**Avocado**	**Psittaciformes (C,E)**
Black locust	Budgerigars (E)
Clematis	Budgerigars (E)
Crown vetch	Budgerigars, Cockatiels, Lovebirds (C)
Dieffenbachia	Canaries(E)
Foxglove	Canaries (E)
Lily of the valley	Pigeons (C,E)
Lupine	Canaries (E)
Oleander	Budgerigars Canaries (E)
Parsley	Ostriches, (C) Ducks (E)
Philodendron	Budgerigars (E)
Poinsettia	Budgerigars (E)
Rhododendron	Budgerigars (E)
Virginia Creeper	Budgerigars (E)
Yew	Pheasants, (E) Canaries (C)

C, clinical report; E, experimental.

## 17 Drugs (diclofenac and barbiturates)

To date, few studies in the literature address drug intoxication due to accidental or iatrogenic ingestion, with most available research focusing on poisoning cases in wild bird populations. The two most frequently reported toxicities involve Diclofenac and Barbiturates. Diclofenac is a non-steroidal anti-inflammatory drug (NSAID) that is commonly used for pain relief and management of inflammation in humans and animals. However, in recent years, diclofenac has been associated with significant toxicity and mortality in certain bird species, particularly scavenging birds such as vultures (83). Diclofenac toxicity in birds primarily affects the kidneys, thus leading to renal failure and visceral gout (84). When birds consume tissues of livestock or other animals treated with diclofenac, the drug is metabolized in their bodies, thus causing the accumulation of toxic metabolites. These metabolites interfere with the normal functioning of the kidneys, thus leading to renal tubular necrosis and impaired uric acid excretion (84, 85). Elevated levels of uric acid in the bloodstream result in visceral gout, which is a condition characterized by the deposition of uric acid crystals in various organs (84, 85). Diclofenac toxicity has had devastating consequences for vulture populations in some regions (86). In South Asia (specifically in India, Nepal, and Pakistan), the use of diclofenac in livestock has led to a severe decline in vulture populations (86–89). Vultures that feed on the carcasses of diclofenac-treated animals suffer from renal failure and succumb to the toxic effects of the drug. The population decline of vultures has had significant ecological and environmental implications, affecting carcass removal and sanitation and disrupting the ecological balance (85, 89). The diagnosis of diclofenac intoxication in birds can be challenging, especially in the early stages when clinical signs may not be apparent. However, if there is a suspicion of diclofenac toxicity based on the bird's exposure history or in presenting symptoms, bloodwork to assess renal function and to investigate abnormalities (such as elevated uric acid levels) should be performed (70). Other blood parameters, such as liver enzymes, electrolytes, and complete blood count, may also be evaluated (7, 10, 90). Treatment of diclofenac intoxication in birds primarily involves supportive care to manage symptoms and to support renal function (10).

Barbiturates are a class of sedative and hypnotic drugs that depress the central nervous system. They have been widely used in veterinary medicine for anesthesia, euthanasia, and the treatment of certain medical conditions. However, barbiturate intoxication in birds can occur when they are exposed to or ingest these drugs in excessive amounts (10, 91). Barbiturate intoxication in birds can have profound effects on their central nervous system and overall health. Common symptoms and effects of barbiturate toxicity in birds may include central nervous system (CNS) and respiratory depression, cardiovascular effects [such as decreased heart rate, hypotension (low blood pressure), and poor circulation), and gastrointestinal symptoms such as vomiting, regurgitation, or diarrhea (10, 92, 93). Avian scavengers have been most frequently affected by barbiturate intoxication, especially Eurasian griffon vultures (*Gyps fulvus*) (92, 93). Diagnosing barbiturate intoxication in birds can be challenging, as it requires a thorough assessment of the bird's exposure history, clinical signs, and laboratory tests. Diagnostic tests may include bloodwork to assess organ function, such as liver and kidney function, as well as a complete blood count. Barbiturates may be detected in blood or tissue samples by using specific analytical methods (93). For other toxicities, supportive care aims to alleviate symptoms and maintain the bird's overall health. This may involve providing supplemental oxygen, fluid therapy to maintain hydration, and nutritional support.

## 18 Hypervitaminosis

Hypervitaminosis refers to a condition in which an excessive amount of vitamins accumulates in the body, thus leading to toxic effects. Although vitamins are essential for bird health, an overabundance of certain vitamins can be harmful. Fat-soluble vitamins, such as vitamins A and D, are more likely to cause toxicity when overdosed (94).

## 19 Hypervitaminosis D

In birds, hypervitaminosis D can occur for various reasons, including excessive supplementation and diet imbalances. Vitamin D exists in various forms, with vitamin D3 and vitamin D2 being the most significant. Although mammals treat these two forms of vitamin D equally in terms of biological activity, many bird species differentiate between vitamin D3 and D2. Vitamin D2, which is naturally found in plants as ergocalciferol, is only approximately one-tenth as effective as vitamin D3 in these avian species (95). Hypervitaminosis D can disrupt calcium balance, thus leading to hypercalcaemia. Hypercalcaemia can lead to the development of soft tissue mineralization, as well as nephrocalcinosis, visceral gout, and urate nephrosis. Birds affected by hypercalcaemia may exhibit clinical signs such as depression, anorexia, vomiting, polyuria and polydipsia, joint pain, and muscle weakness (54). As toxic levels of vitamin D3 can be transferred to the embryo, this can increase embryo mortality (54, 96–98). This scenario can subsequently cause soft tissue calcification, kidney damage, or mineralization of organs (99). Different species appear to have different needs and sensitivities to vitamin D3. Macaw, African gray parrots, and cockatiels seem to be more susceptible to vitamin D toxicity (96, 100, 101). Currently, there is a lack of established optimal and toxic levels of vitamin D3 in the diet for companion birds. The collation of information about the bird's diet, including vitamin D supplementation or exposure to foods high in vitamin D, can help in identifying potential sources of excessive intake. However, caution should be exercised when extrapolating vitamin D3 requirements from poultry to other species of birds (95, 98). Diagnosing hypervitaminosis D in birds can be challenging, as it requires a thorough assessment of the bird's diet, clinical signs, and laboratory tests (102). As hypercalcaemia in birds is most often physiological and associated with increased estrogen secretion and production of calcium-binding proteins (95), careful consideration should be given when interpreting results. Moreover, blood tests may be conducted to measure the levels of vitamin D and to assess calcium and phosphorus levels (102). The calcium concentration should be adjusted to the total protein or albumin concentrations (102). Radiographs or imaging studies may be performed to evaluate bone health and to detect any abnormal calcification. Treatment consists of supportive care to manage symptoms and to support renal function (48). Furosemide treatment is recommended for cases of persistent hypercalcaemia following diuresis with crystalloid fluids (102). Furthermore, calcitonin treatment is recommended for severe and/or persistent hypercalcaemia in mammals, as it effectively and swiftly reduces elevated calcium levels; however, this use in birds has not been validated (96).

## 20 Hypervitaminosis A

Hypervitaminosis A is a condition that occurs when birds have excessive levels of vitamin A in their bodies (103). Vitamin A plays a crucial role in various physiological processes, including vision, growth, and immune function. However, excessive vitamin A intake may result in toxicity and associated health complications. Overdosing on vitamin A supplements or providing excessive amounts of foods high in vitamin A, such as liver, can contribute to the development of clinical signs of an overdose. In rats, a long-term ingestion of modest excesses of vitamin A may contribute to fracture risks (104). Bone lesions have been described in chickens and ducks given large doses of vitamin A, with reduced cortical thickness and common fractures being observed (105, 106). Additionally, young birds with vitamin A intoxication can suffer from osteodystrophy and hypertrophy of parathyroid glands. They may exhibit swollen or reddened eyes, conjunctivitis, and excessive tearing or clouding of the cornea. Moreover, the skin can develop a dark orange color, becoming prone to dryness and cracking (107). Diagnosing hypervitaminosis A in birds can be challenging, as it requires a thorough assessment of the bird's diet, clinical signs, and laboratory tests. The collection of information about the bird's diet, including vitamin A supplementation or high vitamin A-containing foods, can help in identifying potential sources of excessive intake (54). Blood tests may be conducted to measure the levels of vitamin A and to assess liver and renal function. Even if vitamin A and carotenoid concentrations were measured in plasma and liver tissue in birds (108). there are no validated reference ranges for most species. Histopathological examinations of tissues can also aid in the diagnosis, excluding other concomitant pathologies. Additionally, the treatment and prevention of hypervitaminosis A in birds involve several strategies, as the discontinuation of the administration of vitamin A supplements or reducing the intake of vitamin A-rich foods is essential to prevent further toxicity. Providing supportive care, including proper nutrition and hydration, as well as addressing specific symptoms, can also aid in the recovery of affected birds (10, 54).

## 21 Discussion

Toxicoses in birds can arise from various substances and can have severe consequences on their health and wellbeing. It is crucial for bird owners, veterinarians, and researchers to be aware of potential toxic substances and their effects on avian species. Prompt recognition and diagnosis of toxicoses are essential for effective treatment and management. Furthermore, ongoing research and education regarding avian toxicoses are essential to improve our understanding of the subject, as well as enhance diagnostic techniques and develop more effective treatment strategies. By increasing awareness and implementing preventative measures, we can minimize the occurrence of toxicoses in pet birds and promote their overall health and welfare.
